# Shotgun Proteomics Identifies Serum Fibronectin as a Candidate Diagnostic Biomarker for Inclusion in Future Multiplex Tests for Ectopic Pregnancy

**DOI:** 10.1371/journal.pone.0066974

**Published:** 2013-06-24

**Authors:** Jeremy K. Brown, Katarina B. Lauer, Emily L. Ironmonger, Neil F. Inglis, Tom H. Bourne, Hilary O. D. Critchley, Andrew W. Horne

**Affiliations:** 1 Medical Research Council Centre for Reproductive Health, The University of Edinburgh, Queen’s Medical Research Institute, Edinburgh, United Kingdom; 2 Moredun Proteomic Facility, Moredun Research Institute, Pentlands Science Park, Bush Loan, Penicuik, Midlothian, United Kingdom; 3 Queen Charlotte’s and Chelsea Hospital, Imperial College National Health Service Trust, London, United Kingdom; Moffitt Cancer Center, United States of America

## Abstract

Ectopic pregnancy (EP) is difficult to diagnose early and accurately. Women often present at emergency departments in early pregnancy with a ‘pregnancy of unknown location’ (PUL), and diagnosis and exclusion of EP is challenging due to a lack of reliable biomarkers. The objective of this study was to identify novel diagnostic biomarkers for EP. Shotgun proteomics, incorporating combinatorial-ligand library pre-fractionation, was used to interrogate pooled sera (n = 40) from women undergoing surgery for EP, termination of viable intrauterine pregnancy and management of non-viable intrauterine pregnancy. Western blot was used to validate results in individual sera. ELISAs were developed to interrogate sera from women with PUL (n = 120). Sera were collected at time of first symptomatic presentation and categorized according to pregnancy outcome. The main outcome measures were differences between groups and area under the receiver operating curve (ROC). Proteomics identified six biomarker candidates. Western blot detected significant differences in levels of two of these candidates. ELISA of sera from second cohort revealed that these differences were only significant for one of these candidates, fibronectin. ROC analysis of ability of fibronectin to discriminate EP from other pregnancy outcomes suggested that fibronectin has diagnostic potential (ROC 0.6439; 95% CI 0.5090 to 0.7788; P>0.05), becoming significant when ‘ambiguous’ medically managed PUL excluded from analysis (ROC 0.6538; 95% CI 0.5158 to 0.7918; P<0.05). Fibronectin may make a useful adjunct to future multiplex EP diagnostic tests.

## Introduction

Ectopic pregnancy occurs when the conceptus implants and develops outside the uterine cavity, with the vast majority of cases occurring in the Fallopian tube [1], [2]. It complicates 1–2% of pregnancies, causing significant maternal morbidity and occasional mortality [3], [4]. Diagnosis of ectopic pregnancy continues to present a major challenge, with patients often asymptomatic or presenting with non-specific symptoms that do not readily differentiate ectopic pregnancy from intrauterine pregnancy. Whilst in many cases, an ectopic pregnancy will be detected by transvaginal ultrasonography at the first clinic visit [5], transvaginal ultrasonography is often inconclusive and an initial diagnosis of “pregnancy of unknown location” (PUL) is made [6]. In patients with a PUL, subsequent diagnosis of ectopic pregnancy relies on the serial measurement of serum human chorionic gonadotrophin levels (and, in some centers, progesterone), together with follow-up transvaginal ultrasonography [3], [4], [7]. This approach is expensive, resource intense and significantly delays diagnosis and management of ectopic pregnancy, increasing the risk of tubal rupture and life-threatening intra-abdominal haemorrhage [8]. There remains an unmet need for a diagnostic test capable of identifying ectopic pregnancy at first clinical presentation, with a blood test or similar minimally invasive test being the ultimate goal [7], [9].

Nearly 30 candidate serum biomarkers of ectopic pregnancy have been identified based on their etiological roles in ectopic pregnancy [10]–[16]. However, this hypothesis-driven approach has been slow to generate new candidates and has yet to deliver a diagnostic test that has been fully validated for use in the clinic. Unbiased global genomic and proteomic approaches offer a quicker route to novel biomarker discovery. For example, microarray analysis of the gene expression profile of endometrium from patients with ectopic pregnancy has yielded a number of promising leads [17]–[19], with serum levels of activin B appearing to differentiate ectopic pregnancy from both viable and non-viable intrauterine pregnancy, albeit in a relatively small population [17]. Nevertheless, it is the ability to interrogate the proteome of biological specimens directly, and with ever increasing degrees of sophistication, that holds the greatest promise for rapid identification of novel biomarkers of ectopic pregnancy. A recent global proteomic study, employing multidimensional separation together with trypsin digestion and quantitative MS/MS to compare sera from women with ectopic pregnancy versus viable intrauterine pregnancy, has almost doubled the list of previously published potential biomarkers [20]. However, when women present with pain and/or bleeding and a PUL there are more than two potential final outcomes. Here, we describe the identification of a candidate biomarker of ectopic pregnancy employing a commercial combinatorial ligand library (ProteoMiner™, BIO-RAD) to facilitate the identification for novel low-medium abundance biomarkers using a shotgun proteomics approach [21]. Initially, we compared sera collected retrospectively from women with known pregnancy outcomes of ectopic pregnancy, viable intrauterine pregnancy and non-viable intrauterine pregnancy. We then tested candidate biomarkers of ectopic pregnancy informed by our proteomic data in a separate, well-defined, prospective case-control study that included every possible PUL final outcome, not only ectopic pregnancy and viable intrauterine pregnancy.

## Results

### Sample Separation and PMF of Differentially Expressed Bands

**Figure 1:**
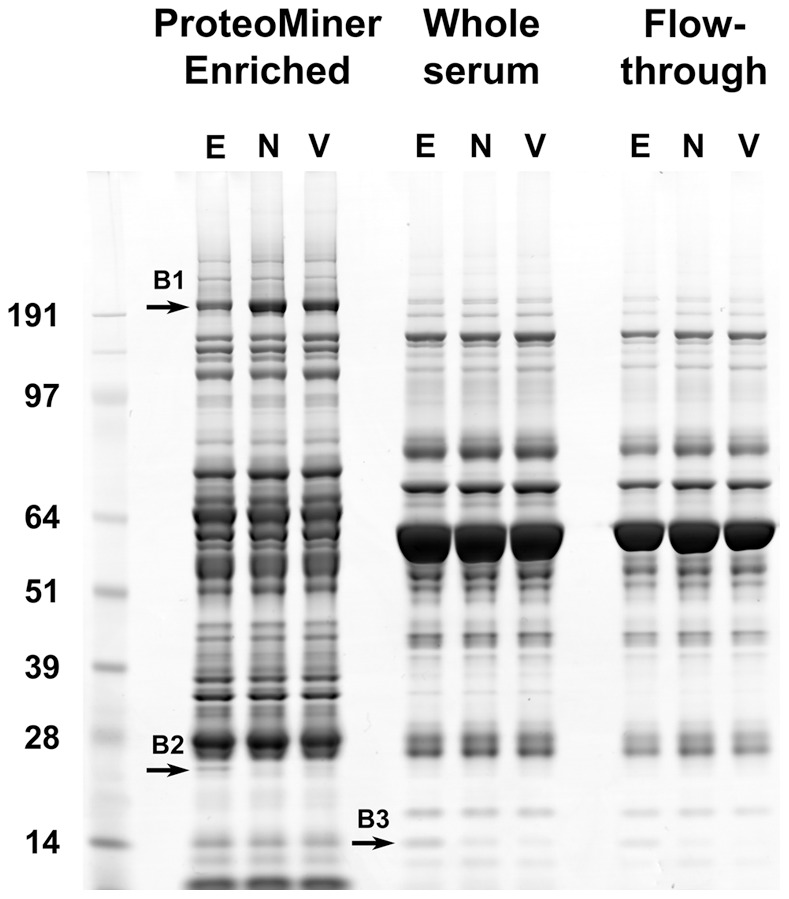
1D SDS-PAGE of ProteoMiner™ affinity-purified serum, alongside the original whole sera and flow-through, from women undergoing surgical management of EP (E), surgical management of NVIUP (N) and surgical termination of VIUP (V). Note the increased complexity and reduction in high abundance proteins in the enriched fractions. Three bands (B1, B2 and B3) appeared differentially expressed and were submitted for PMF.

Pooled sera collected from women during surgical management of ectopic pregnancy (EP: confirmed at surgery and by pathology; n = 15); nonviable intrauterine pregnancy (NVIUP: USS-confirmed intrauterine gestational sac with yolk sac and/or embryo without cardiac activity seen prior to uterine evacuation: n = 10); and termination of viable intrauterine pregnancy (VIUP: USS-confirmed intrauterine gestational sac with an embryo with cardiac activity seen prior to uterine evacuation; n = 15), were pre-fractionated using ProteoMiner™ spin-columns and separated by SDS-PAGE, alongside whole sera and unbound flow-through from the spin-columns (Figure 1). Lanes loaded with ProteoMiner™ enriched fractions contained more protein bands than those loaded with whole sera or unbound flow-through from the spin-columns. By contrast, only minor differences were observed between lanes loaded with whole sera and flow-through, suggesting that the vast majority of serum proteins that are normally visible by SDS-PAGE saturate the ProteoMiner™ spin-columns and were preferentially depleted from the enriched fractions. Comparison of the three patient groups revealed distinct bands in ProteoMiner™ enriched (B1 and B2) and whole serum (B3) fractions that appeared to be differentially expressed in the EP group. B1 appeared to be reduced in the EP group, whereas B2 and B3 both appear to be more abundant in the EP group. All three bands were excised and submitted for PMF by MALDI-TOF. Definitive results were obtained for B1, which was identified as FN1 (Mascot Score 377; 52/88 tryptic peptides assigned to FN1; 34.0% sequence coverage; Predicted MW 266.1 KDa). Positive identities were subsequently obtained for B2 (C reactive protein (CRP): Mascot score 414; 7 unique tryptic peptide spectra containing four or more contiguous ‘y’ or ‘b’ ions; 27.7% sequence coverage; Predicted MW 25.0 KDa) and B3 (hemoglobin subunit beta (HBB): Mascot score 650; 10 unique tryptic peptide spectra containing four or more contiguous ‘y’ or ‘b’ ions; 75.5% sequence coverage; Predicted MW 16.0 KDa) using LC-ESI-MS/MS. Predicted and observed molecular weights were consistent in all three cases (Figure 1).

### Shotgun LC-ESI-MS/MS

**Figure 2:**
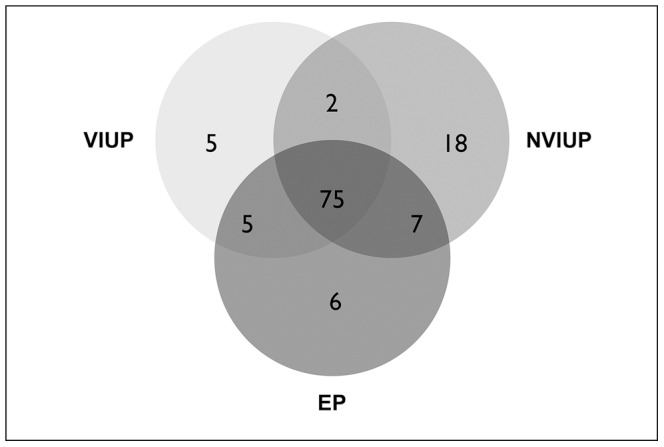
Pooled sera from women undergoing surgical management of EP, surgical management of NVIUP and surgical termination of VIUP were affinity-purified using ProteoMiner™ spin-columns and separated by 1D SDS-PAGE, prior to trypsin digestion and LC-ESI-MS/MS. Interrogation of the SwissProt database produced a total of 118 non-redundant high confidence identities.

Shotgun LC-ESI-MS/MS analysis was performed on ProteoMiner™ enriched fractions following SDS-PAGE. After deconvolution of raw MS/MS data, interrogation of the SwissProt database produced a total of 1412 non-redundant identities (Table S1). Filtering of these results, such that protein identities were based on the assignment of at least two unique peptide spectra containing a series of four or more contiguous ‘y’ or ‘b’ ions, reduced this number to 118 high confidence identities (Figure 2). Of these: 75 were common to all three patient groups; a further 12 were present in the EP group and either the VIUP or NVIUP groups; 6 were unique to the EP group; 2 were uniquely absent from the EP group; and a further 5 and 18 were unique to the VIUP and NVIUP groups respectively. Following exclusion of potential environmental contaminants (various cytokeratin isoforms and trypsin), five biomarker candidates were selected for follow-up screening on the basis of their apparent differential expression in pooled sera from the EP group (MASP1, HBB and ADIPO) or the VIUP group (PSG3 and PSG4) (Table 1). CRP and FN1 were also included for follow-up screening on the basis of SDS-PAGE results (Figure 1). However, shotgun proteomics (Table 1) and analysis of CRP levels in individual sera (data not shown) using an existing clinical assay (Clinical Chemistry unit, Lothian University N.H.S. Trust, Royal Infirmary of Edinburgh) did not support further analysis of CRP as a biomarker candidate.

**Table 1:** Candidate biomarkers identified by Shotgun LC-ESI-MS/MS.

Accession	Name	Mascot score EP	Mascot score NVIUP	Mascot score VIUP	PeptidesEP	PeptidesNVIUP	PeptidesVIUP	SC [%]EP	SC [%]NVIUP	SC [%]VIUP
MASP1_HUMAN	Mannan-binding lectin serine protease 1	151.32	73.31	15.42	4	2	1	8.30	3.72	2.15
HBB_HUMAN	Hemoglobin subunit beta	144.49	62.83	0	7	1	0	29.93	8.84	0
FN1_HUMAN*	Fibronectin	3548.02	4499.04	4025.33	164	205	233	39.19	45.47	43.76
ADIPO_HUMAN	Adiponectin	123.88	158.21	101.96	5	6	5	23.37	21.72	15.57
PSG3_HUMAN	Pregnancy-specific beta-1-glycoprotein 3	0	0	110.01	0	0	5	0.00	0.00	6.78
PSG4_HUMAN	Pregnancy-specific beta-1-glycoprotein 4	0	27.84	110.37	0	1	5	0.00	2.63	7.40
CRP_HUMAN*	C-reactive protein	377.27	289.61	333.71	21	7	10	27.68	20.98	27.23

LC-ESI-MS/MS data derived from pooled sera obtained from women prior to surgical management of definite ectopic pregnancy (EP), surgical management of definite nonviable intrauterine pregnancy (NVIUP) and surgical termination of definite viable intrauterine pregnancy (VIUP) were used to interrogate the SwissProt database and the results summarised in terms of: Mascot score; number of identified tryptic peptides; and sequence coverage (SC [%]). Following exclusion of potential environmental contaminants, six candidate biomarkers were selected for follow-up screening on the basis of their apparent differential expression in pooled sera from ectopic patients or women with viable intrauterine pregnancies.

* FN1 and CRP were included on the basis that they were identified to be down-regulated in pooled sera from ectopic patients by 1DGE (B1 and B2 Fig.1).

### Western Blot Validation of LC-ESI-MS/MS Data

**Figure 3:**
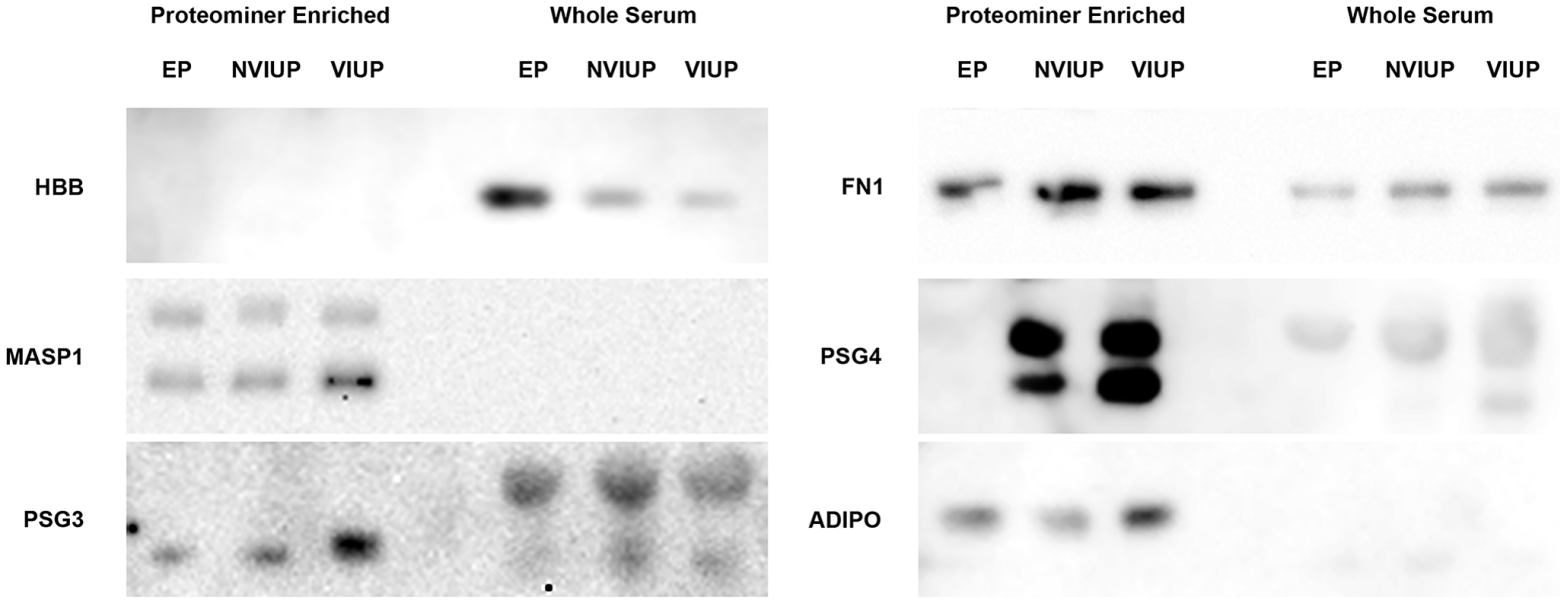
Western blots of pooled whole sera and ProteoMiner™ affinity-purified serum from women undergoing surgical management of EP, surgical management of NVIUP and surgical termination of VIUP were probed with antibodies specific for proteins identified by LC-ESI-MS/MS (Table 1).

Western blots of pooled whole sera and ProteoMiner™ enriched fractions, probed with specific antibodies to proteins identified by mass spectrometry (Tables 1), are shown in Figure 3 (full length images of individual blots are provided in Figure S1). With the exception of HBB, pre-fractionation with ProteoMiner™ affinity spin-columns resulted in enrichment of target proteins. However, as predicted by SDS-PAGE (Figure 1. B3) and shotgun proteomics (Table 1), HBB appeared to be increased in whole serum from the EP group and was selected for follow-up analysis. In contrast to the shotgun proteomics data (Table 1), western blot analysis of ProteoMiner™ enriched fractions did not support increased levels of MASP1 in serum from the EP group (Figure 3). Instead, it supported a reduction in the 35 KDa MASP1 light chain in both the EP and NVIUP groups. Given this conflict with the shotgun proteomics data and technical difficulties encountered with detecting MASP1 in whole serum, MASP1 was excluded from follow-up analysis. Western blots for the remaining four candidates (PSG3, PSG4, FN1 and ADIPO) produced results that were consistent with the shotgun proteomics data. However, the signal-to-noise ratio obtained for specific PSG3 labelling was not sufficient for reliable measurement of PSG3 in individual whole sera using western blot, and this candidate was not carried through to further validation studies.

**Figure 4:**
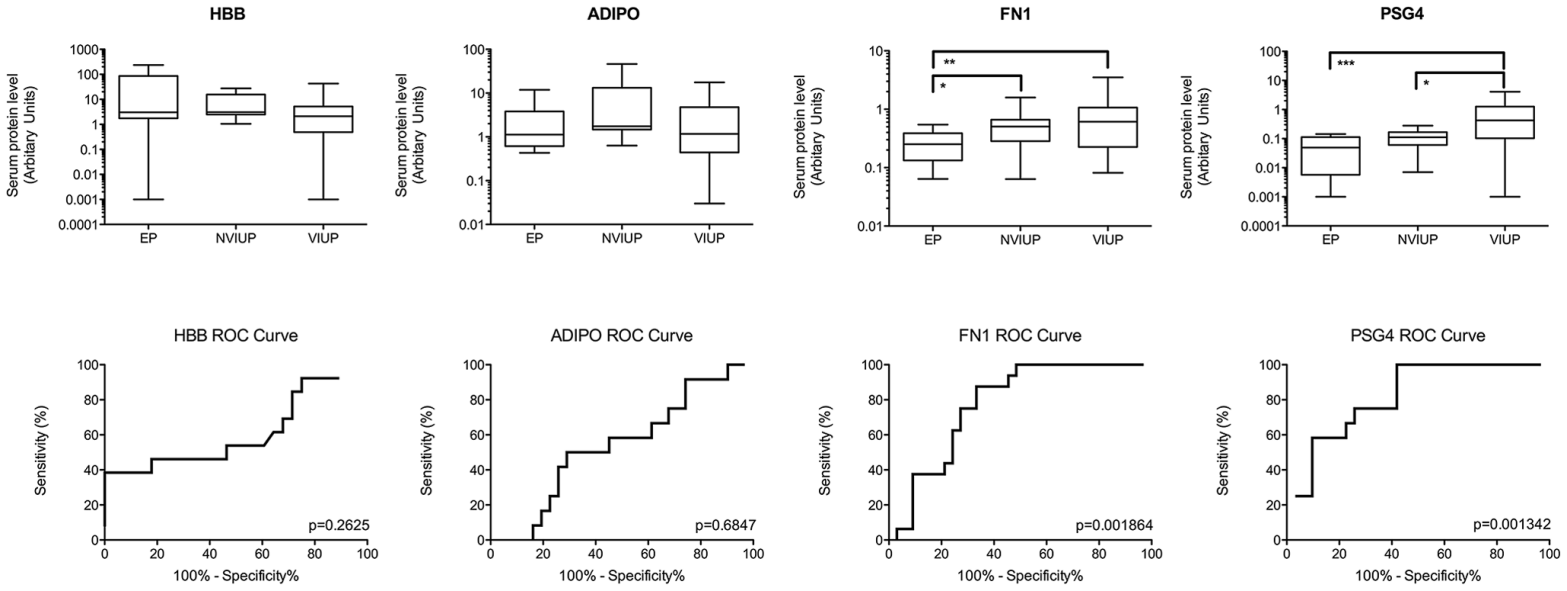
Western blots of individual whole sera from women undergoing surgical management of EP, surgical management of NVIUP and surgical termination of VIUP were probed with antibodies specific for: HBB; ADIPO; FN1; and PSG4. Integrated density values of specifically labelled bands were normalised against a positive control (pooled VIUP serum). ROC curves were generated for ectopic vs non-ectopic patient groups.

Western blots of individual whole sera from the retrospective cohort were probed with antibodies specific for selected biomarker candidates: HBB, ADIPO, FN1 and PSG4 (Figure 4). Nonparametric one-way ANOVA (Kruskal-Wallis) detected significant differences in FN1 (P<0.01) and PSG4 (P<0.0001) levels between patient groups. Post-test non-parametric analysis identified significant decrease in FN1 and PSG4 levels in sera from the EP group compared to the VIUP group (Mann Whitney test: P<0.01 and <0.001 respectively). Additionally, there was a significant difference in FN1 levels between the EP and NVIUP groups (Mann Whitney test: P<0.05). In contrast, PSG4 appears to be a marker of viable intrauterine pregnancy, rather than EP, and was significantly reduced (Mann Whitney test: P<0.05) in the NVIUP group, when compared to the VIUP group. When receiver operating characteristic (ROC) analysis of the ability of each candidate to discriminate EP patients from both VIUP and NVIUP patients was performed, both FN1 (Area under the ROC curve 0.7765; 95% CI 0.6489 to 0.9041; P<0.01) and PSG4 (Area under the ROC curve 0.8185; 95% CI 0.6909 to 0.9462; P<0.01) appeared to have diagnostic value when applied to the retrospective cohort (Figure 4).

### ELISA Analysis of Serum FN1 and PSG4

**Figure 5:**
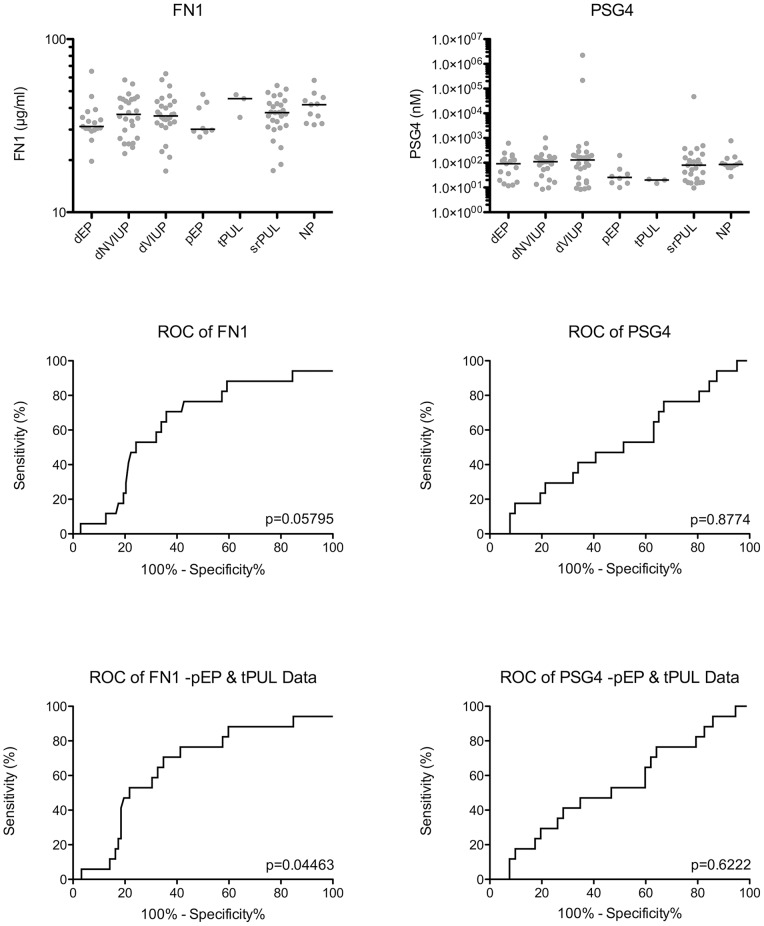
FN1 and PSG4 ELISA of serum collected at first presentation of women with a PUL, categorized according to final pregnancy outcome: dNVIUP (n = 26); dVIUP (n = 28); dEP (n = 17); NP (n = 11); srPUL (n = 27); pEP (n = 8); and tPUL (n = 3). ROC curves were generated for ectopic vs non-ectopic patient groups. Analysis was repeated after ambiguous medically treated PUL outcomes (pEP and tPUL) were excluded (-PUL Data).

FN1 and PSG4 were selected for further validation in a larger prospective cohort prospective cohort (n = 120): sera were obtained from women (age 18–45 years) at the time of their first clinical presentation with pain and/or bleeding prior to initial diagnosis of a PUL. All women were monitored until their discharge from hospital and their final pregnancy outcomes were classified (Table 2: Table S2), as per the recent consensus statement [22]. FN1 (Intra assay CV 1.08%; Inter Assay CV 6.29%; 150 ng/ml sensitivity 90%) and PSG4 (Intra assay CV 13.1%; Inter Assay CV 4.84%; 2 nM sensitivity 97%) competitive ELISAs were developed and validated for use on human serum samples. Nonparametric one-way ANOVA (Kruskal-Wallis) did not detect significant differential expression of FN1 or PSG4 in the dEP group within the prospective cohort (Figure 5: P>0.05). However, ROC analysis (Figure 5) of the ability of FN1 to discriminate dEP patients from all the other groups combined suggested that FN1 does have diagnostic potential (Area under the ROC curve 0.6439; 95% CI 0.5090 to 0.7788; P>0.05), becoming significant when pEP and tPUL are excluded from the analysis (Area under the ROC curve 0.6538; 95% CI 0.5158 to 0.7918; P<0.05). ROC analysis did not support the use of PSG4 as a biomarker of ectopic pregnancy in this cohort.

**Table 2:** Patient recruitment: 120 patients with an initial diagnosis of a PUL were recruited to the study and grouped according to final pregnancy outcomes.

Group	Inclusion criteria	hCG (mU/ml)	Age (years)	BMI	n
dVIUP	Definite viable intrauterine pregnancy: TVUSS confirmation of intrauterine gestationalsac with yolk sac and embryo with cardiac activity.	6844±2017	28±1	26±2	28
dNVIUP	Definite nonviable intrauterine pregnancy: USS confirmation of intrauterine gestationalsac with yolk sac and/or embryo without cardiac activity seen prior to uterineevacuation. Confirmed by histopathology.	4022±1904	32±1	27±1	26
dEP	Definite ectopic pregnancy: intervention prompted by adnexal mass on TVUSS or byabnormal rise in serum hCG levels and confirmed at surgery and by histopathology.	1151±238	29±1	25±1	17
NP	Not pregnant: positive home pregnancy test result subsequently not confirmedby serum hCG measurement.	<5	26±2	27±3	11
srPUL	Spontaneously resolving PUL: PUL with spontaneous resolution of serum hCG levels.	428±114	32±1	28±1	27
tPUL	Treated persistent PUL: abnormal rise in serum hCG levels but no adnexal mass or IUsac seen on TVUSS after monitoring, managed medically with methotrexate.	400±188	32±4	28±5	3
pEP	Probable ectopic pregnancy: inhomogenous adnexal mass or extrauterine sac-likestructure on TVUSS managed medically with methotrexate.	597±200	33±1	25±1	8

## Discussion

Using a shotgun proteomics approach that incorporated combinatorial ligand library pre-fractionation [21] to normalize the dynamic range of serum protein concentrations, rather than the more established approach of using panels of monoclonal antibodies to deplete high abundance serum proteins [23], we have identified FN1 as a potential adjunct diagnostic biomarker for EP. Although our study is not the first to employ a shotgun proteomics approach to ectopic pregnancy biomarker discovery [20], [24], it is the first to use the technique to compare sera from patients with ectopic pregnancy, viable and non-viable intrauterine pregnancy (Figure 2). The ability to differentiate an ectopic pregnancy from a non-viable intrauterine pregnancy, and other final PUL outcomes, will be critical to the success of any future ectopic pregnancy diagnostic test. This study is also the first, to our knowledge, that has tested the utility of potential ectopic pregnancy biomarker candidates using prospectively collected first presentation sera from an independent cohort of women with a complete range of PUL final clinical outcomes (Figure 5).

Like biomarker candidates previously identified by our group [17], [25], both FN1 and PSG4 showed considerable promise as biomarkers of ectopic pregnancy when assessed in the context of a relatively simple retrospectively sampled cohort of women with ectopic pregnancy, viable intrauterine pregnancy or non-viable intrauterine pregnancy (Figure 4). However, ELISA analysis of sera collected from women at their first clinical presentation with a PUL (Figure 5) revealed a less marked reduction in FN1 and PSG4 serum levels in women who required surgical intervention for an ectopic pregnancy (Figure 4). Nevertheless, ROC analysis suggested that serum FN1 levels may be useful in differentiating ectopic pregnancy from other pregnancy outcomes (Figure 5), with its diagnostic value becoming significant (Area under the ROC curve 0.6538; 95% CI 0.5158 to 0.7918; P<0.05) when the medically managed PUL outcomes, for which a definitive final diagnosis was not possible (pEP and srPUL), were excluded from the analysis (Figure 5). This reduction in diagnostic value, when applied to a ‘real world’ cohort of patients demonstrates the importance of study design.

A previous proteomics study [20] identified a disintegrin and metalloprotease-12 (ADAM-12) as a potential ectopic pregnancy biomarker, which has since been validated in a sera from a larger cohort of women with ectopic pregnancy or viable intrauterine pregnancy, collected at the time of presentation at emergency departments with pelvic pain and/or bleeding and a positive pregnancy test, and appears to be able to differentiate ectopic pregnancy from viable intrauterine pregnancy with a good degree of sensitivity and specificity [26]. We did not identify ADAM-12 as a potential biomarker for ectopic pregnancy in our study, possibly due to differences in the sensitivity of the systems used and/or pre-fractionation methods, or possibly due to the inclusion of our non-viable intrauterine pregnancy control group. However, when we recently attempted to test ADAM12 as a biomarker for ectopic pregnancy in the same cohort of patients with a PUL used in the current study [27], we could not replicate the findings of Rausch et al [26]. Crucially, serum levels of ADAM12 were virtually identical in women with srPUL and dEP, groups that require very different clinical management. Gestational age is likely to be a key factor in ADAM12 levels, as it rises exponentially from around week 5 of the first trimester [28]. Therefore, it seems possible that the lower levels of ADAM12 we reported [27] reflect the relatively early gestational age of our prospectively collected first presentation PUL cohort. There is also debate as to the specificity of ADAM-12 with regard to differentiating ectopic pregnancy from outcomes other than viable intrauterine pregnancy [29] and its clinical utility remains unclear at this time. When compared directly, FN1 appears to have greater potential that ADAM12 in identifying women who require surgical intervention for an ectopic pregnancy at their first presentation with a pain and/or bleeding and a PUL.

It was interesting that both our proteomics study, and the previous proteomics study [20], [24], identified differences in serum levels of pregnancy-specific beta-1-glycoproteins (specifically PSG4/9) consistent with older published data [30], [31]. However, in our study this finding became no longer significant when we included dNVIUP and/or other PUL groups in our analysis. It is possible that this is due to a difference in the assays used to detect PSG, however we believe it is also a function of and demonstration of the importance of a study design including these additional clinical categories.

While we believe that our findings are important, we acknowledge that our prospectively collected cohort is small and FN1 does not appear to have sufficiently high specificity and sensitivity as a diagnostic biomarker in isolation. The diagnosis of ectopic pregnancy requires both very high specificity and sensitivity given that a false negative could lead to serious morbidity and potential mortality. Similarly, a false positive result could lead to the termination of a viable pregnancy and we agree with other investigators in the field that the best future test will almost certainly be a multiplexed assay specific for a panel of biomarkers [7]. Recent analysis of the performance of a multiple marker test based on four existing biomarkers candidates (progesterone, VEGF, inhibin A and activin A) has produced encouraging results [32]. Nonetheless, there remains the key question of differentiating ectopic pregnancy from PUL outcomes other than viable intrauterine, and such a test might benefit from inclusion of FN1. Furthermore, our findings suggest that further scrutiny of changes in components of the pathways involved in regulation of serum FN1 levels and/or sequestration of FN1 during pregnancy [33] may provide additional, more robust candidate biomarkers of ectopic pregnancy. It will be of interest to determine whether changes in FN1 levels associated with ectopic pregnancy are more marked in plasma, compared to serum where the majority of FN1 is sequestered during the coagulation process.

## Materials and Methods

### Ethical Approval

Ethical approval for this study was obtained from the Lothian Research Ethics Committee (LREC 04/S1103/20 and 09/S1103/39), with informed written consent obtained from patients.

### Setting

Patients were recruited from the Pregnancy Support Centre at the Royal Infirmary of Edinburgh from March 2010 to January 2011.

### Patient Groups

Retrospective cohort (n = 40): sera were obtained from women (aged 18–45 years) at time of surgery: undergoing surgical termination of viable intrauterine pregnancy (USS-confirmed intrauterine gestational sac with an embryo with cardiac activity seen prior to uterine evacuation); surgical management of nonviable intrauterine pregnancy (USS-confirmed intrauterine gestational sac with yolk sac and/or embryo without cardiac activity seen prior to uterine evacuation, n = 10); and surgical management of ectopic pregnancy (confirmed at surgery and by pathology, n = 15).

Prospective cohort (n = 120): sera were obtained from women (age 18–45 years) at the time of their first clinical presentation with pain and/or bleeding prior to initial diagnosis of a PUL. All women were monitored until their discharge from hospital and their final pregnancy outcomes were classified (Table 1), as per the recent consensus statement [22].

### Sample Pre-fractionation and One Dimensional Gel Electrophoresis

Pooled sera from the retrospective cohort were pre-fractionated using ProteoMiner™ affinity spin-columns (BioRad laboratories, Hertfordshire, UK) following the manufacturers instructions. 5 µg of pooled whole sera, ProteoMiner™ flow-through and ProteoMiner™ enriched fractions from each sample were separated by one dimensional gel electrophoresis (1DGE: NuPAGE 4–12% Bis-Tris gels (Invitrogen)). Gels were stained with Imperial Protein stain (Thermo Scientific, Rockford, USA) as per the manufacturers instructions.

### Targeted Peptide Mass Fingerprinting

Where 1DGE revealed gross differences between patient groups in the instructive cohort, differentially expressed bands were submitted for peptide mass fingerprinting (PMF). Selected bands were digested with trypsin and PMF was performed by Matrix-assisted laser desorption/ionization time-of-flight mass spectrometry (MALDI-TOF: Ultraflex™ II, Bruker Daltonics, Bremen, Germany). Band identity was determined by database searches of SwissProt using the Mascot search algorithm (http://www.matrixscience.com: using “homo sapiens” as a taxonomical search parameter; mass tolerance of ±0·15 Da; and fixed modification = Cys-carbamidomethylation). Where MALDI-TOF results were not definitive, PMF was performed by liquid chromatography electrospray ionization tandem mass spectrometry (LC-ESI-MS/MS) as previously described [34]. Following deconvolution of raw MS/MS data, database searches of SwissProt, using “homo sapiens” as a taxonomical search parameter, were performed via ProteinScape® software (Bruker) and the Mascot search algorithm (Matrix Science). Proteins identities were assigned higher confidence if two or more of their predicted unique tryptic peptides, with MS/MS spectra containing four or more contiguous ‘y’ or ‘b’ ions, were detected.

### Shotgun LC-ESI-MS/MS

1DGE was performed on ProteoMiner™ enriched fractions from each patient group in the instructive cohort. Lanes corresponding to EP, NVIUP and VIUP patient groups were divided into 27 slices, subjected to standard in-gel trypsinolysis and LC-ESI-MS/MS as described above.

### Western Blot Analysis

1DGE was performed on ProteoMiner™ enriched pooled sera and whole sera (pooled and individual samples). Gels were blotted onto Immobilon-P membrane (Millipore, Livingston, UK) using a Transblot-SD (Bio-Rad Laboratories, Hemel Hempstead, UK). After blocking for 30 minutes in staining buffer (Tris buffered saline +0.5% Tween-20+2% fat-free milk powder), blots were incubated for 2 hours with: 1 µg/ml of rabbit anti-Mannan binding lectin serine protease 1 (Abcam, Cambridge, UK); 30 ng/ml of rabbit anti-FN1 (Sigma); 1 µg/ml of rabbit anti-Pregnancy specific beta 1 glycoprotein 3 (Abcam); 1 µg/ml of control rabbit IgG (Abcam); a 1 in 1500 dilution of mouse polyclonal anti-Pregnancy specific beta 1 glycoprotein 4 (Abcam); a 1 in 1500 dilution of normal mouse serum (Sigma); 1 µg/ml of monoclonal mouse anti-Adiponectin IgG_1_ (Abcam); 1 µg/ml of monoclonal mouse anti-Hemoglobin subunit beta IgG_1_ (Abcam); or 1 µg/ml of control Mouse IgG_1_ (Sigma). Blots were washed (six three minute changes of Tris buffered saline +0.5% Tween-20) and incubated for 1 hour with the 20 ng/ml of appropriate Alkaline phosphatase (ALKP) conjugated secondary antibodies (Stratech Scientific, Newmarket, UK). Following washing, specific ALKP labelling was detected using CDP-STAR Star (Boehringer-Mannheim, Bracknell, UK). Images were acquired using a VersaDoc™ Imaging System (Bio-Rad Laboratories) and specific Chemiluminescence was measured using ImageJ [35], and expressed as relative to a positive control included in each gel (pooled VIUP serum).

### FN1 ELISA

Purified human FN1 (Sigma) was biotinylated using an EZ-Link® Sulfo-NHS-Biotin kit (Thermo Shandon; Runcorn, UK). MICROLON® 200 96-well microplates (Greiner Bio-One, Stonehouse, UK) were coated overnight at 4°C with 50 µl/well of rabbit anti-human FN1 (Sigma) diluted to 50 ng/ml in 0.2 M NaHCO_3_/Na_2_CO_3_ buffer (pH 9.6). Plates were washed (six washes with 0.15 M NaCl +0.05% Tween-20) and blocked for 1 hour at 21°C with 100 µl/well of ELISA buffer (Tris buffered saline +0.5% Tween-20+4% bovine serum albumin). Purified human FN1 standards (150 ng/ml –10 µg/ml) and serum samples (diluted 1 in 10 in ELISA buffer) were incubated for 10 minutes with equal volumes of biotinylated-FN1 (1 µg/ml). Plates were washed and incubated for 1 hour at 21°C with 50 µl/well of biotinylated-FN1/standards and biotinylated-FN1/serum samples. Plates were washed and incubated for 30 minutes with ultrasensitive streptavidin-peroxidase polymer (Thermo Fisher Scientific, Cramlington, UK) diluted to 1 in 10000 in ELISA buffer. After washing, plates were incubated with 50 µl/well of SureBlue™ peroxidase substrate (KPL, Inc., Maryland, USA) for 15 minutes before the reaction was stopped with 100 µl/well of 0.1 M HCl and the absorbance of the product measured at 450 nm.

### PSG4 ELISA

MICROLON® 200 96-well microplates were coated overnight at 4°C with 50 µl/well with 250 nM PSG4 peptide (sc-240791 P: Santa Cruz Biotechnology, Inc. Heidelberg, Germany) in 0.2 M NaHCO_3_/Na_2_CO_3_ buffer (pH 9.6). Plates were washed and blocked for 1 hour at 21°C with 100 µl/well of ELISA buffer. PSG4 peptide standard (256 nM –2 nM) and serum samples were incubated for 10 minutes with equal volumes of 0.2 µg/ml of goat anti-PSG4 (Santa Cruz Biotechnology, Inc.). Plates were washed and incubated for 1 hour at 21°C with 50 µl/well of goat anti-PSG4/standards and goat anti-PSG4/serum samples. After washing, plates were incubated for 1 hour at 21°C with 50 µl/well of 200 ng/ml of biotinylated donkey anti-goat (Abcam). Plates were washed and incubated for 30 minutes with ultrasensitive streptavidin-peroxidase polymer diluted to 1 in 10000. After washing, plates were developed as described above.

### Statistical Analysis

Statistical analyses, ELISA standard curve formulae and receiver operating characteristic (ROC) curves were generated using Prism (GraphPad Software, La Jolla, USA).

## Supporting Information

Figure S1 **Full length images of western blots of pooled whole sera and ProteoMiner™ affinity-purified serum from women undergoing surgical management of EP, surgical management of NVIUP and surgical termination of VIUP were probed with antibodies specific for proteins identified by LC-ESI-MS/MS (** **Table 1** **).**
(DOC)

Table S1 (XLS)

Table S2 (XLS)
