# Supplementary material for: Shotgun Proteomics Identifies Serum Fibronectin as a Candidate Diagnostic Biomarker for Inclusion in Future Multiplex Tests for Ectopic Pregnancy
Source: PLoS One. 2013 Jun 24;8(6):e66974. doi: 10.1371/journal.pone.0066974 (PMC3691233; doi:10.1371/journal.pone.0066974)

Supplemental Figure 1: Full length images of western blots of pooled whole sera and ProteoMiner™ affinity-purified serum from women undergoing surgical management of EP, surgical management of NVIUP and surgical termination of VIUP were probed with antibodies specific for proteins identified by LC-ESI-MS/MS (Table 1).

1. HBB: Predicted molecular weight: 16 kDa


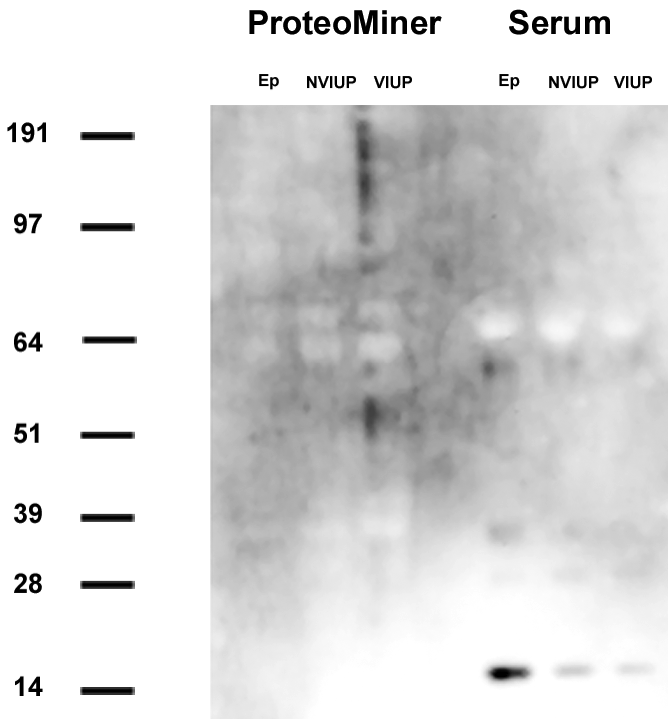


1. MASP1: Predicted molecular weight: 48 kDa


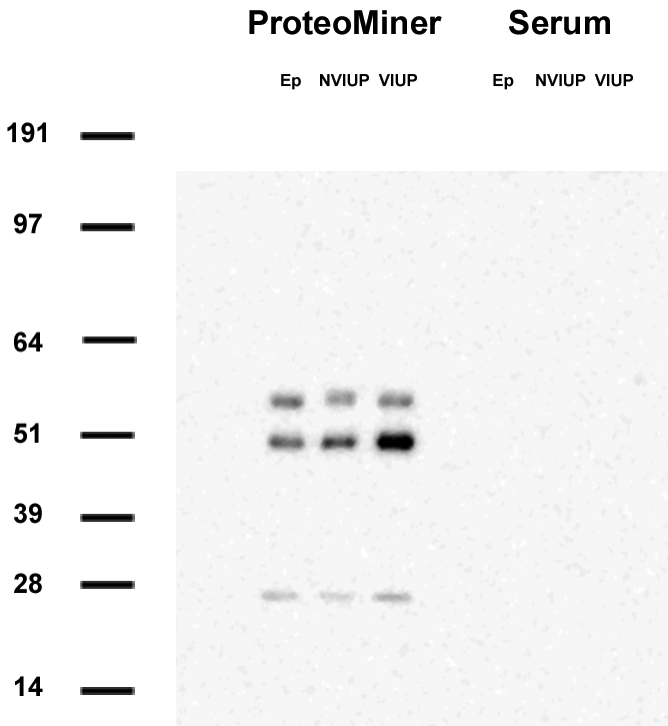


1. PSG3: Predicted molecular weight: 48/60 kDa


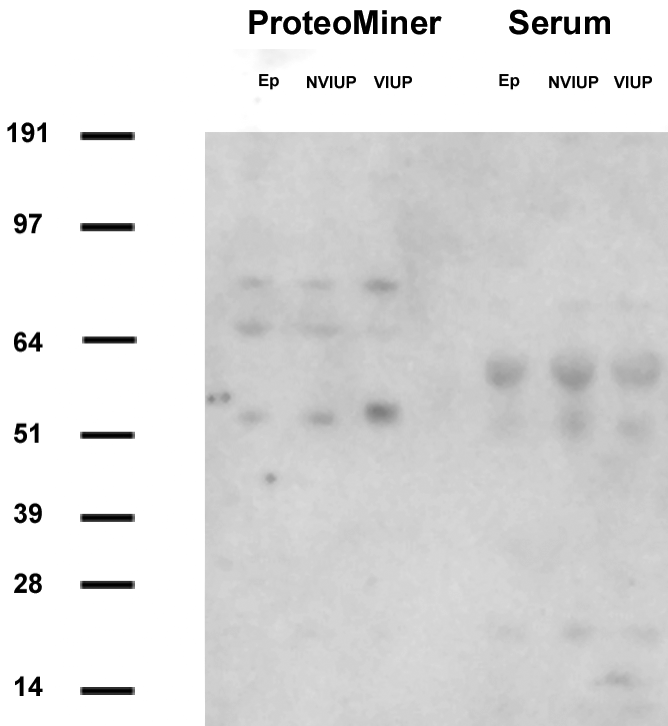


1. FN1: Predicted molecular weight: 265 kDa


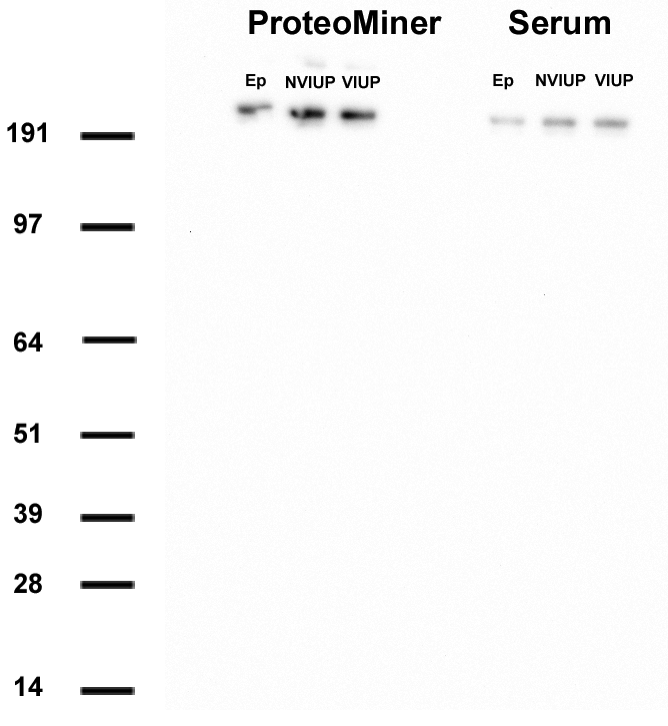


1. PSG4: Predicted molecular weight: 48/60 kDa


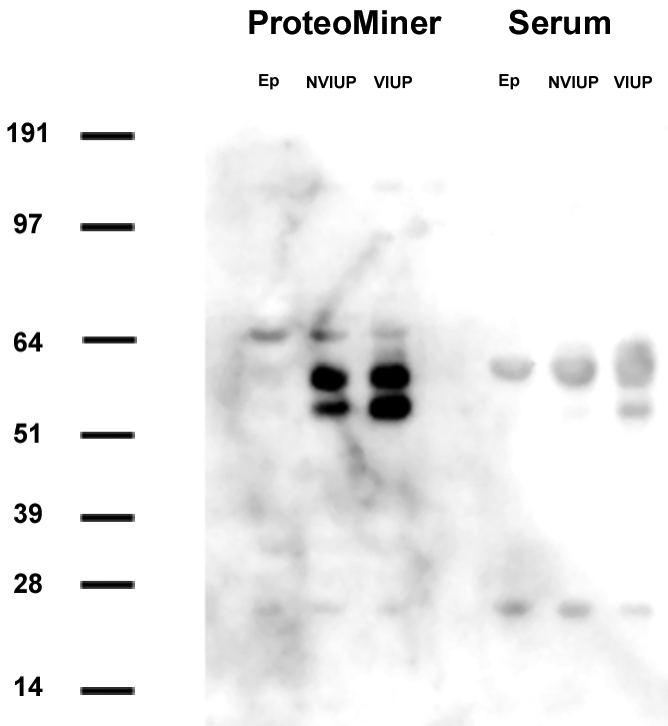


1. ADIPO: Predicted molecular weight: 26/30 kDa


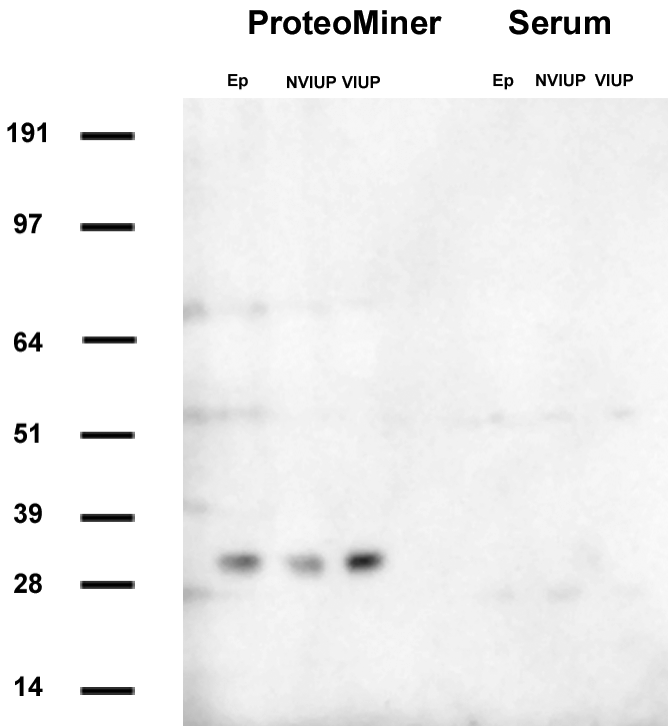

Supplement: Figure S1 — Full length images of western blots of pooled whole sera and ProteoMiner™ affinity-purified serum from women undergoing surgical management of EP, surgical management of NVIUP and surgical termination of VIUP were probed with antibodies specific for proteins identified by LC-ESI-MS/MS ( Table 1 ). (DOC) [file pone.0066974.s001.doc]
